# Report of Cases

**Published:** 1914-02

**Authors:** R. R. Kime

**Affiliations:** Atlanta, Ga.


### REPORT OF CASES.

By R. R. Kime, M. D., Atlanta, Ga.

I—Carbolic Acid Poisoning—1 Tablespoonful—Recovery.
II—Tonsilitis followed by Nephritis and Convulsions.
III—Fulminating Appendicitis—Abscess, Peritonitis—Cesorean Section.

CASE I. Mrs. W., age 50 years, married, suffering with Intestinal Toxænia and mild nephritis, had been in bed several days and just convalescing, being subject to constipation, for which purified petroleum oil had been prescribed in tablespoonful doses.

A pint bottle of Carbolic Acid was sitting by the bottle of oil, and the acid was given pure. I was called as soon as the mistake was recognized, and ordered over the phone a half tablespoonful of alcohol, and in a few minutes one teaspoon of

Spirits Camphor, both to be taken without water, as they happened to have them in the house. I ordered my machine, dressed quickly and went immediately to the home, about ten squares away. I found patient unconscious and could not swallow, having convulsive jerkings, gave a hypodermic of apomorphia, finding my stomach tube was not likely to work, despatched runners to drug store and near-by sanatorium for stomach tubes and more olive oil.

The hypodermic did not act, and it was about 40 or 45 minutes before I succeeded in getting a pint of olive oil with camphor into the stomach, and while I was waiting for it to have some effect, Dr. Niles arrived. He pumped out the oil and camphor. Then we threw a pint of plain oil into the stomach and let it remain for some time, then drew off the excess. She began to breathe better and circulation improved. Dr. Niles was compelled to leave at this time.

Soon patient could begin to swallow, and we gave one tablespoon of olive oil every 15 minutes, later put the oil farther apart and gave milk of magnesia, half tablespoonful with 4 drops essence bitter almond aromatic, every 1 to 2 hours. Used enema of oil to move bowels.

No water was allowed the first 12 hours. Half teacupful of oilve oil was given each afternoon and morning, also oil enemas to move bowels. Egg albumen was the first nourishment given, later liquid peptonoids. Patient lived on liquids for ten days, then began to take small amount of soft diet. She seems to have made a complete recovery. No trouble in swallowing solid food. No evidence of any stricture, and is now in her usual health and no evidence of any kidney lesion remaining. I believe the essential factor in saving this case was not using any water, not even to wash out the stomach, as advised by many.

I would never use water in carbolic acid poisoning, unless I could not get oil. Then I would want alcohol with the water.

Patients can stand an immense amount of carbolic acid if you have alcohol, camphor and olive oil to use, and keep water away. Even though the phenol is combined with alcohol and camphor, it must not be left in the stomach, as water will precipitate the camphor and set free the phenol, so oil should be used to wash out the stomach. I have known of one tablespoon of camphorated phenol to kill a man inside of two hours, and I was told the stomach was washed out.

Olive oil is best, as it combines perfectly with the camphor and carbolic acid, cotton seed oil is not so good, as it does not combine so well. I know some authorities advise against oil in carbolic acid poisoning.

My experience with camphorated phenol and olive oil has convinced me of its usefulness.

Lister was first to pour carbolic acid, pure, into abscessed cavities, with excellent results. Dr. Powell, of New York, was first to demonstrate alcohol as an antidote. He washed out abscessed cavities with pure carbolic acid, followed with alcohol.

CASE II. Female, age 14, previous health good, measles at 6 years, no history of previous kidney trouble.

Called April 11, 1913, found patient with follicular tonsilitis. Making but the one visit, expecting them to report the next morning for further investigation. Patient returned to school. Ten days followed, was examined at school by the school physician, and pronounced well. The day following was taken with convulsions about 1 p. m. Saw her soon after, but no evidence of anything wrong that I could see, except a slight elevation of pulse and tongue slightly coated. Prescribed laxative and intestinal antiseptic.

About one hour later was called again, patient having second convulsion. Found pulse 85 to 90, some slight nervousness, temperature yet normal. Prescribed Tr. Ver. Ver. Nor. bromide sodium with alkaline diuretic. About one hour later I was called again, patient having third convulsion, pulse and nervousness slightly increased, no elevation of temperature, pupils normal, patient rational, no pain nor tenderness could be found on physical examination. I told parents I felt confident the convulsions were due to intestinal toxæmia and want of elimination by the kidneys, ordered the medicines rushed up and bowels moved. In less than one hour, they called my residence, stating patient was having another convulsion. When I

arrived home, I decided to go and stay long enough to see if she was really having convulsions. I found patient with pulse about 110, temperature one degree above normal, resting very well. In about 20 minutes she had a hard convulsion, remaining unconscious afterward, with difficult breathing. I at once gave veratrium hypodermically. She remained unconscious and had another long, hard convulsion in a short time. I then gave hypodermic of morphia and atrophia. Patient looked so serious I advised parents to call help.

At this time there were cases of cerebro-spinal meningitis in the city. Dr. A. was first to arrive and diagnosed cerebro-spinal meningitis. Shortly Dr. B. arrived and was inclined to same diagnosis and went for spinal puncture apparatus. In the meantime, Dr. C. arrived and concluded we had a case of status epilepticus with unfavorable prognosis. Later Dr. B. returned, and spinal puncture failed to secure any fluid. As patient was doing very well, treatment was continued.

Patient remained unconscious throughout the night, having but one convulsion. Next morning Dr. B. and I gave patient pilocarpin hypodermically and put her in hot pack, after which she regained consciousness for the first time. Pilocarpin and hot packs were repeated once and twice daily as needed until elimination was established by the bowels and kidneys. The first specimen of urine showed pus red blood cells, hyaline and granular casts, and a trace of albumen, later pus casts. The patient had no more convulsions after the first night and gradually improved. With dieting and treatment for several months the kidney lesion gradually cleared up.

If this case had died the first night it would have been classed as cerebro-spinal meningitis, but as it turned out I am certain it was a case of nephritis due to follicular tonsilitis.

Dr. J. B. Murphy gives us some very definite clinical data on metastatic infections and says the regularity of their occurrence indicates the variety of germ producing the primary and secondary infection. He states: "Streptococcus infection metastasis in joints occurs in 24 to 72 hours. Gonococcus in 18 to 22 days in the majority of cases. Pneumonococcus in 11 to 15

days and Grip infections in about the same time. Typhoid fever during third or fourth week."

As a clinician and teacher, he has no equal.

CASE III. Mrs. S—, 20 years, married eight months, gave history of having had two attacks of appendicitis in the last three years, also a miscarriage three months previous, with one monthly flow since, and having a second flow at present attack.

Nine days previous to operation ate a hearty supper, followed with raw pears—was that night taken with severe pain diagnosed by attending physician as appendicitis, and so treated, with improvement. One week later, Friday afternoon, patient again ate a hearty supper with raw pears and had an attack during the night—severe pain, rapid pulse, high temperature, continuing until I saw her Sunday at 4 p. m. I found evidences of suppurative appendicitis and peritonitis, abdomen so distended and tender could not map out anything by pelvic examination, but advised immediate operation as best chance to save life of patient. She was moved immediately to the Georgia Baptist Hospital and abdomen opened at border of right rectus muscle. Uterus was not curetted because we could not map out condition of pelvic organs and the condition of the patient demanded quick work. This decision saved the life of the patient, as you will see later. To have undertaken to curette uterus the patient would have died on the table before completing the work. We found omentum matted to the intestines and intestines adherent to each other and uterus. Broke up adhesions, lifting up cæcum entering an abscess, but no appendix found—followed line of cleavage, opening another abscess in region of right ovary, then following up and separating intestines, liberated a third collection of pus in Douglas' Pouch. We then had separated a soft fluctuating mass the size of a child's head in center of abdomen, dark color, looking and feeling more like an ectopic pregnancy than a pregnant uterus. Made a median incision into the mass and found a pregnancy of four or five months. We immediately emptied the uterus and carried gauze drain wet with alcohol through cervix, leaving one end loosely folded in uterine cavity and closed incision with catgut in less than twelve minutes. Searched again, short time only, for appendix, and failing to find it, introduced tubular and cigarette drains into each of abcess cavities (3) and closed abdominal wall up around drains, all coming out at one point. Gave hypodermoclysis of salt solution immediately following operation. Patient kept in Fowler's position and proctoclysis by Murphy method used.

Abdominal drains gradually pulled up, i. e., 1-2 to 1 inch each day. As soon as abdominal cavity was thoroughly walled off Iodine solution (Tr. Iodine 1-2 to 1 drachm, alcohol and sterile water aa 4 ounces), a small amount was poured into drainage cavity each day. Removed cigarette gauze drains entirely on fourth day. Gauze strips saturated with Iodine solution was loosely packed around drainage tubes, removing, cleansing, and replacing tubes each day after fifth or sixth day—gradually shortening tubes as cavity filled from bottom. To protect the skin from irritating, discharge gauze saturated with an oil mixture (Thynol Iodid 1 drachm, Bismuth Sub Nitrate 2 drachms, olive oil 4 ounces), was placed around wound. Later, this oil mixture was used in and around wound until there was a clean, shallow, granulating surface with no pus, then wound and skin were approximated by adhesive strips.

The Bismuth and Iodide-oil mixture was not poured into cavity until you could see and reach bottom of cavity, to sponge out any accumulation—but even before that it can be used on gauze loosely packed into wound. One invariable precaution used is to keep external surface of wound well open, making it funnel shaped. Wounds handled in this way almost invariably heal without a second operation and with far less scar tissue.

This patient is well, menstruating regularly, so far without hernia or a second operation. Large doses of quinine, diuretics, etc., were used in the Proctoclysis, Burnham's Soluble Iodine by mouth in this case.

Later, I hope to present a paper on the management of these fulminating cases, also the use of quinine, Iodine and Hexamethylenamine in abdominal and pelvic surgery.

